# Efficacy of Intratonsillar Steroid Injection for Reducing Palatine Tonsil Size in Tonsillar Hypertrophy: A Randomized Controlled Trial

**DOI:** 10.1055/a-2798-2798

**Published:** 2026-09-29

**Authors:** Arunrat Dumkliang, Ekachai Dumkliang

**Affiliations:** 1 Department of Otorhinolaryngology, Thungsong Hospital, Thungsong, Nakhon Si Thammarat, Thailand; 2 Drug Delivery System Excellence Center, Department of Pharmaceutical Technology, Faculty of Pharmaceutical Sciences, Prince of Songkla University, Hat Yai, Songkhla 90110, Thailand

**Keywords:** obstructive sleep apnea (OSA), palatine tonsil, tonsillar hypertrophy, steroid, normal saline solution(NSS)

## Abstract

**Introduction:**

Tonsillectomy is a common treatment for obstructive sleep apnea (OSA) and remains one of the most frequently performed surgical procedures worldwide. However, postoperative morbidity is a persistent clinical concern. Intratonsillar steroid injection may offer a non-surgical alternative by reducing the size of hypertrophic palatine tonsils.

**Objective:**

this study aims to evaluate the efficacy and safety of intratonsillar steroid injections compared to intralesional injections of normal saline solution (NSS) in tonsillar hypertrophy.

**Study design:**

Prospective study (Randomized control trial)

**Materials and Methods:**

In this randomized controlled trial, 19 participants (38 tonsils) were assigned to four groups receiving intratonsillar injections of triamcinolone acetonide (TA, 40 mg/mL, 1 mL) or NSS (1 mL). Nine participants received multiple injections at weeks 0, 4, and 8; ten received a single injection. Tonsil size was measured before each injection to assess the clinical efficacy of reducing palatine tonsil size.

**Results:**

There was a significant reduction in tonsil size after steroid injections, with a statistically significant difference observed only in the multiple steroid injection group (p-value = 0.04). The steroid injection group experienced slightly more pain compared to the NSS injection group, however, no severe adverse effects were observed in either group.

**Conclusion:**

Intratonsillar steroid injections effectively reduced the size of palatine tonsils compared with NSS injections. Repeated steroid injections were more effective than a single injection.

## Introduction

The tonsils, part of Waldeyer's ring, are primarily composed of lymphoid tissue and play a crucial role in generating immunity by capturing and destroying pathogens that enter the body through the upper respiratory tract. Repeated inflammation and infection in the upper respiratory tract are significant factors affecting the balance of lymphoid tissue proliferation and involution, leading to tonsillar hypertrophy. 1 This condition may result in obstructive sleep apnea (OSA), affecting both children and adults. In children, OSA can impair physical, cognitive, and emotional development, while in adults it increases the risk of cardiovascular complications such as hypertension, ischemic heart disease, heart failure, arrhythmias, and cerebrovascular accidents. 2 3 4 5

Tonsillectomy is a common treatment for OSA and is one of the most frequently performed surgical procedures worldwide. However, postoperative morbidity remains a significant clinical concern, with reported complications including pain, poor oral intake, dehydration, bleeding, airway compromise, and, rarely, death. 6 7 8 Current evidence suggests that intranasal corticosteroids may significantly reduce adenoid size, offering a less invasive treatment option compared to adenoidectomy. 9 10 However, intranasal corticosteroids have not been effective in reducing the size of enlarged palatine tonsils, likely due to their anatomical location and washout by saliva.

Previous clinical studies have shown that intralesional steroid can lead to complete resolution of lymphoid hypertrophy at the hard palate without serious adverse events. 11 However, the efficacy of intratonsillar steroid injection for reducing size of tonsillar hypertrophy in human has not been reported. Only one study has demonstrated that intratonsillar fluticasone propionate injection is effective in reducing palatine tonsil size in a rabbit model. The purpose of this study was to determine whether intratonsillar steroid injection reduces the size of palatine tonsils in humans, building on previous evidence of the efficacy and safety of triamcinolone acetonide (TA) for intraoral lesion injection. 11 12 13 14

## Materials and Methods

### Subjects

This study was approved by Nakhon Sri Thammarat Provincial health ethics committee (No. NSTPH 0202564), and informed consent was obtained from each participant. This prospective study, randomized controlled trial involved healthy volunteers age 18-40 years with bilateral tonsillar hypertrophy of grade 2+ or greater, who had no indication for tonsillectomy, and no contraindication for steroid injection. Participants were randomly assigned to one of four groups using a single-blind design, which included single or multiple intratonsillar injection of TA or NSS.

### Endoscopic Examination

The tonsils were anesthetized and gag reflex was reduced with 10% xylocaine spray. A tongue depressor was placed on the midline of the tongue, and a 0-degree rigid endoscope was introduced into the oral cavity to photograph the oropharynx and palatine tonsils. The endoscopic evaluation aimed to ensure easy and safety detectability of the tonsils, correlating real tonsil size with subjective assessment. 15 16 Tonsillar hypertrophy was assessed using Brodsky's scale, which grades airway obstruction as follows: 0 (no obstruction), 1+ (<25%), 2 + (25%-50%), 3+ (50%-75%), and 4+ (>75%). 17

### Intratonsillar Injection

After applying 10% lidocaine spray and photographing of oropharynx, 1 mL of TA (40 mg/mL) was injected into the center of one palatine tonsil ( Fig. 1 ) and 1ml of NSS was injected into the center of the other palatine tonsil, using a 25-gauge needle.

**Fig. 1:**
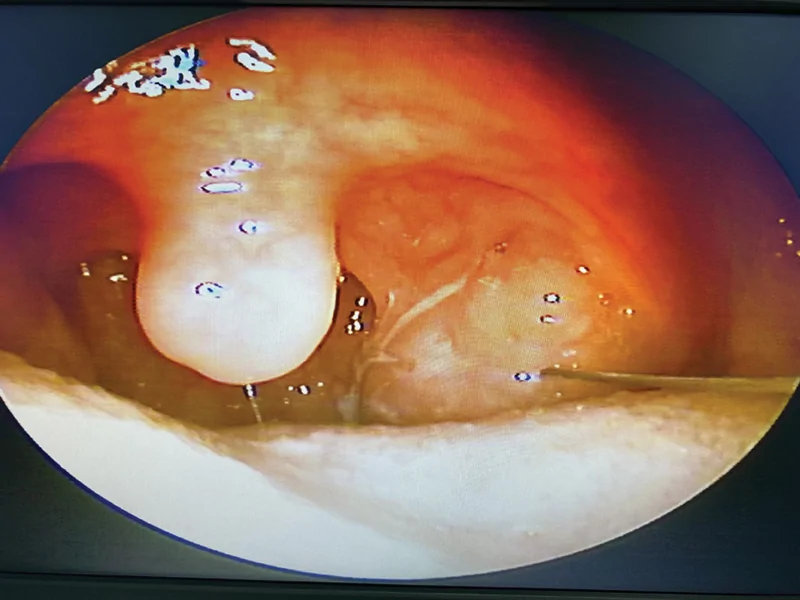
Intratonsillar steroid injection technique.

### Experimental Procedure

Participants were randomly assigned to one of four groups ( Fig. 2 ).

**Fig. 2:**
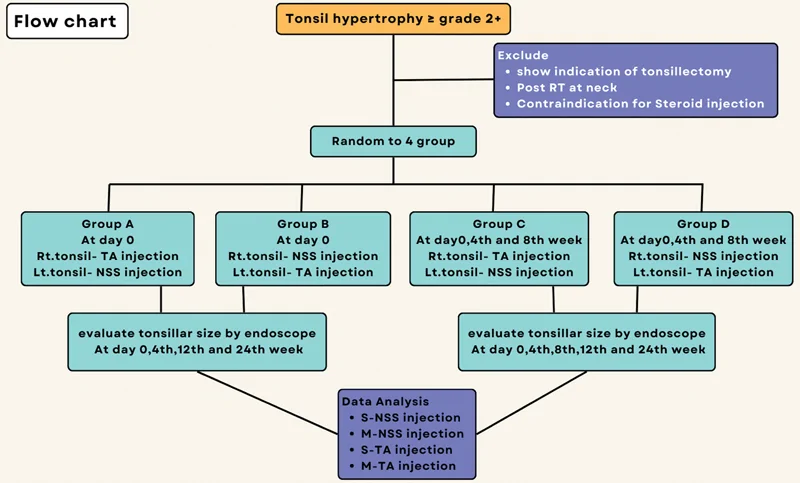
Study flowchart showing participant allocation to the four injection groups.

-Group A: single TA injected to right tonsil and single NSS injected to left tonsil.
-Group B: single TA injected to left tonsil and single NSS injected to right tonsil.
-Group C: multiple TA injected to right tonsil and multiple NSS injected to left tonsil.
-Group D: multiple TA injected to left tonsil and multiple NSS injected to right tonsil.

Groups A and B received single TA and NSS injection on day 0, tonsil size measured at weeks 0, 4, 12, and 24 Groups C and D received multiple TA and NSS injections at week 0,4 and 8, with the tonsil size measured before each injection at week 0, 4, 8, 12 and 24. Exclusion criteria included severe oral disease such as mucositis grade ll, active infection, bleeding tendency and pregnancy. Injections would be discontinued if adverse effects such as infection or hemorrhage occurred following treatment.

### Outcome Measurements

The subjective size of palatine tonsils was assessed to compare the efficacy of single steroid versus single NSS, multiple steroid versus multiple NSS injections and single steroid versus multiple steroid injections. Digital photographs of tonsils were taken for comparison ( Fig. 3 ).

**Fig. 3:**
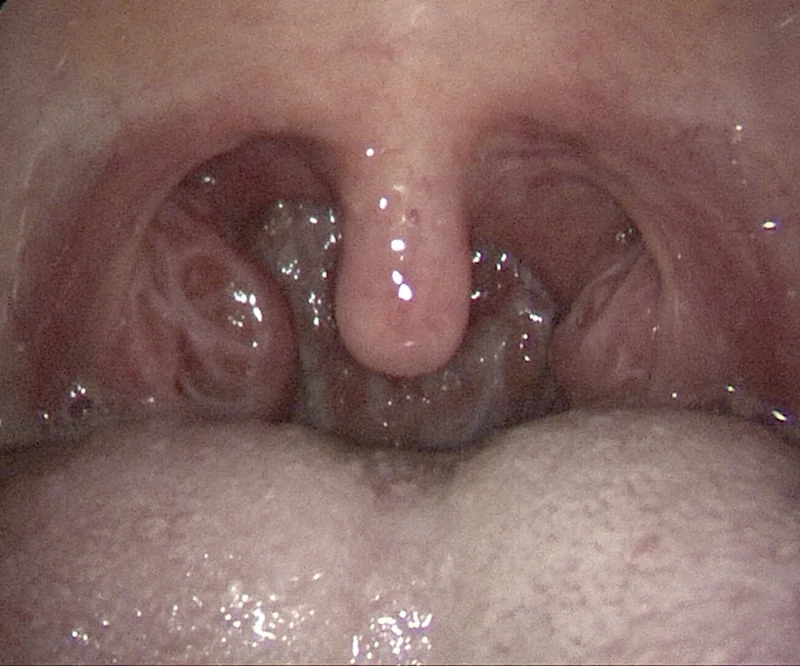
Digital photograph of Group D tonsils at week 24.

Pain was assessed using a numerical rating scale. Patients were asked to assign a numerical score representing the intensity of their pain on a scale from 0-10, with 0 being no pain and 10 being worst imaginable pain. 18

### Statistical Analysis

Data were analyzed statistically. Between-group differences were assessed using one-way ANOVA, and within-group comparisons were performed using the paired t-test. All p-values < 0.05 were considered statistically significant.

## Results

A total of 38 palatine tonsils from 19 participants were included in this study. One participant contributed tonsils to two groups - one tonsil in the TA injection group and the other in the NSS injection group (control). The four study groups were: Single NSS injection (S-NSS), Multiple NSS injection (M-NSS), Single TA injection (S-TA), and Multiple TA injection (M-TA).

The average age of participants in the single injection group was 31.8 ± 2 years, while in the multiple injection group it was 31.6 ± 2.2 years ( Table 1 ). There were only three men in this study: two in the single injection group, and one in the multiple injection group.

**Table 1:** Sex, Age and Tonsil size distribution

	S-NSS	M-NSS	S-TA	M-TA
Sex (N)	10	9	10	9
Male	2	1	2	1
Female	8	8	8	8
Age (year)	31.8 ± 2	31.6 ± 2.2	31.8 ± 2	31.6 ± 2.2
Tonsil size (% airway obstruction)	56.34 ± 15.06	69.37 ± 13.5	55.47 ± 12.39	68.07 ± 6.81
Tonsil grading	3+	3+	3+	3+
Underlying Disease	No	No	No	No

The average percentage of airway obstruction due to tonsillar hypertrophy was 62.30% (range 55-70%). All participants had grade 3+ tonsillar hypertrophy and no underlying diseases.

At 4 weeks, the M-NSS, S-TA, and M-TA groups showed a reduction in tonsil size. The multiple injection groups exhibited a greater reduction in tonsil size compared with the single injection groups. Overall, the M-TA group demonstrated the most significant reduction in tonsil size ( Fig. 4 ).

**Fig. 4:**
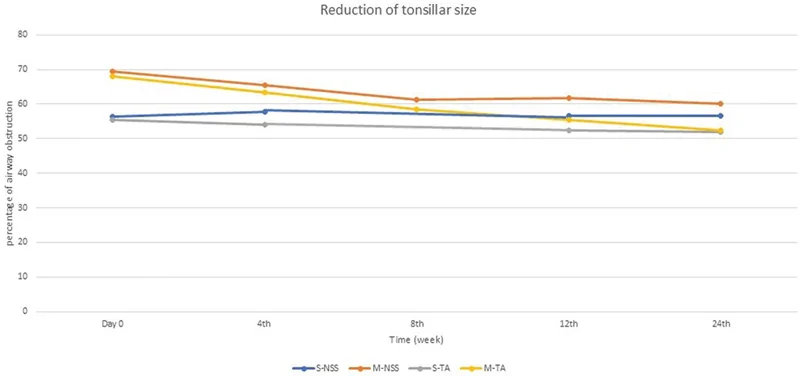
Reduction in tonsil size across the four injection groups over the 24-week follow-up period.

No statistically significant differences were found in the size reduction of tonsils in the S-NSS, M-NSS and S-TA group (p = 0.99, 0.34, and 0.92, respectively) ( Table 2 ). Only the M-TA group showed a statistically significant reduction in tonsils size (p = 0.04) ( Table 2 ), with the greatest reduction observed at the 24-week mark (t = 0.01).

**Table 2:** Sex, Age and Tonsil size distribution

S-NSS					
Time	Average (% airway obstruction)	Variance	ANOVA Source of Variation	*df*	*P-value*
Day 0	56.34	226.93	Between Groups of time	*3*	*0.99*
4 ^th^ week	57.82	187.98	Within Groups of time	*36*	
12 ^th^ week	56.53	90.74			
24 ^th^ week	56.50	87.15	Total	*39*	
**M-NSS**					
**Time**	**Average** **(% airway obstruction)**	**Variance**	**ANOVA** **Source of Variation**	***df***	***P-value***
Day 0	69.37	182.20	Between Groups of time	*4*	*0.34*
4 ^th^ week	65.39	130.51	Within Groups of time	*40*	
8 ^th^ week	61.24	95.11			
12 ^th^ week	61.68	73.74			
24 ^th^ week	60.06	75.62	Total	*44*	
**S-TA**					
**Time**	**Average** **(% airway obstruction)**	**Variance**	**ANOVA** **Source of Variation**	***df***	***P-value***
Day 0	55.43	153.62	Between Groups of time	*3*	*0.92*
4 ^th^ week	54.04	162.93	Within Groups of time	*36*	
12 ^th^ week	52.33	168.62			
24 ^th^ week	51.83	154.73	Total	*39*	
**M-TA**					
**Time**	**Average** **(% airway obstruction)**	**Variance**	**ANOVA** **Source of Variation**	***df***	***P-value***
Day 0	68.67	46.39	Between Groups of time	*4*	*0.04*
4 ^th^ week	63.43	98.16	Within Groups of time	*40*	
8 ^th^ week	58.59	146.47			
12 ^th^ week	55.49	180.61			
24 ^th^ week	52.51	183.63	Total	*44*	

No participants in any group developed adverse effects such as infection or hemorrhage following treatment. The full planned course of injections was completed in all patients, and none developed side effects that necessitated discontinuation of treatment. The pain score in the TA group was slightly higher than in the NSS group (0.78 +- 0.2 vs. 0.30 +- 0.08, respectively). A few participants took a single dose of paracetamol 500 mg for pain relief after injection.

## Discussion

Tonsillar hypertrophy may lead to OSA, affecting both children and adults. 2 3 4 5 Studies have shown that the proliferation rates and concentration of inflammatory mediators in tonsillar cell culture from children with OSA and recurrence infection indicate that lymphoid tissue proliferation in these condition may be regulated by different mechanisms. 19 Goldbart et al. reported increased expression of glucocorticoid receptors in adenotonsillar tissue in pediatric OSA. 20 The number of glucocorticoid receptors may vary depending on factors such as the degree of immunological activation and the stage of tissue proliferation. 21 22

Glucocorticoids have several physiological and pharmacological effects, with one of the most important being their strong suppressive influence on the inflammatory response that causes tissue swelling. 23 24 Evidence suggests that glucocorticoids induce apoptosis of activated T lymphocytes. 23 Recent studies have shown that steroids reduce the size of lymphoid tissue. 9 10 11 25 26 27 Based on this concept, we designed a study to measure the size of palatine tonsils after intratonsillar steroid injection, as intralesional steroid injections have fewer systemic adverse effects than systemic steroid. 14 28 Among available corticosteroids, triamcinolone acetonide (TA) was selected due to its long-acting properties, low water solubility, and high potency for local injection. TA has been used effectively for pain relief from tonsillectomy. 8 29 30 Furthermore, previous studies have demonstrated the efficacy and safety of TA for intraoral lesion injection. 11 12 13 14

Anjomshoaa et al. reported a case of lymphoid hyperplasia at the hard palate, which was treated with weekly TA intralesion injections. The condition resolved completely after four injections and no recurrence was observed during a seven-month follow-up. 11 Kheirandish-Gozal, et al. found that steroids increased cellular apoptosis of *in vitro* tonsillar proliferation in a dose-dependent manner, with fluticasone showing the highest potency. 26 Recently, Do-Yeon et al. demonstrated that intratonsillar steroid injections statistically significantly reduced the size of palatine tonsils in a rabbit model, with repeated injections being more effective than a single injection. 27

The present results are consistent with those of Do-Yeon et al., who demonstrated a significant reduction in tonsil size following TA injection. 27 However, statistically significant differences in size reduction of tonsils were only found in the M-TA group ( *p-value =0.04* ). Do-Yeon, et al used Fluticasone propionate for intratonsillar injection, and their results showed statistically significant reductions in tonsil size with both single and multiple steroid injection compared to the saline injection group. 27 The differing results may be attributable to differences in the potency of the corticosteroid selected.

In the present study, the TA group experienced slightly more pain than the NSS group, possibly because TA is more irritating to tissue. No severe adverse effects were reported in either group, consistent with previous studies showing the safety of TA intraoral lesional injection. 11 12 13 14

The variability in our results may be due to the relatively small number of patients in the study. Therefore, we recommend increasing the number of participants in future studies. In addition, this study was conducted on volunteers with enlarged but otherwise normal tonsils, without symptoms of recurrent tonsillitis or obstructive sleep apnea. Consequently, the effectiveness of this treatment in patients with chronic infection or symptomatic disease (e.g., obstructive sleep apnea) remains uncertain, and further studies involving symptomatic patients are warranted. Furthermore, the development of a device to deliver the drug directly into the tonsils could provide a minimally invasive alternative to tonsillectomy in selected patients.

## Conclusion

Intratonsillar steroid injection effectively reduced the size of the palatine tonsils compared to NSS injections. Repeated steroid injections were more effective than a single injection.
